# *TP53* alterations in primary and secondary Sézary syndrome: A diagnostic tool for the assessment of malignancy in patients with erythroderma

**DOI:** 10.1371/journal.pone.0173171

**Published:** 2017-03-16

**Authors:** Audrey Gros, Elodie Laharanne, Marie Vergier, Martina Prochazkova-Carlotti, Anne Pham-Ledard, Thomas Bandres, Sandrine Poglio, Sabine Berhouet, Béatrice Vergier, Jean-Philippe Vial, Edith Chevret, Marie Beylot-Barry, Jean-Philippe Merlio

**Affiliations:** 1 INSERM U1053, Bordeaux Research in Translational Oncology University Bordeaux, Bordeaux, France; 2 Tumor Bank and Tumor Biology Laboratory, CHU de Bordeaux, Pessac, France; 3 Dermatology Department, CHU de Bordeaux, Bordeaux, France; 4 Pathology Department, CHU de Bordeaux, Pessac, France; 5 Hematology Laboratory, CHU de Bordeaux, Pessac, France; Universitat Zurich, SWITZERLAND

## Abstract

Recent massive parallel sequencing data have evidenced the genetic diversity and complexity of Sézary syndrome mutational landscape with *TP53* alterations being the most prevalent genetic abnormality. We analyzed a cohort of 35 patients with SS and a control group of 8 patients with chronic inflammatory dermatoses. *TP53* status was analyzed at different clinical stages especially in 9 patients with a past-history of mycosis fungoides (MF), coined secondary SS. *TP53* mutations were only detected in 10 patients with either primary or secondary SS (29%) corresponding to point mutations, small insertions and deletions which were unique in each case. Interestingly, *TP53* mutations were both detected in sequential unselected blood mononuclear cells and in skin specimens. Cytogenetic analysis of blood specimens of 32 patients with SS showed a *TP53* deletion in 27 cases (84%). Altogether 29 out of 35 cases exhibited *TP53* mutation and/or deletion (83%). No difference in prognosis was observed according to *TP53* status while patients with secondary SS had a worse prognosis than patients with primary SS. Interestingly, patients with *TP53* alterations displayed a younger age and the presence of *TP53* alteration at initial diagnosis stage supports a pivotal oncogenic role for *TP53* mutation in SS as well as in erythrodermic MF making *TP53* assessment an ancillary method for the diagnosis of patients with erythroderma as patients with inflammatory dermatoses did not display *TP53* alteration.

## Introduction

Epidermotropic cutaneous T-cell lymphomas, *i*.*e*. mycosis fungoides (MF) and Sézary syndrome (SS), account for approximately 65% of cutaneous T-cell lymphomas [1, 2]. Early stages MF (Ia-IIa) are characterized by limited and fixed skin patches or plaques and a low proportion (10 to 20%) of neoplastic T-cells in the upper dermis and epidermis. At advanced stages (IIb-IVb), corresponding to tumor or erythroderma, tumor cells become more abundant and epidermotropism may be lost [2, 3].

Conversely, Sézary syndrome occurs *de novo* at an advanced stage (IV) and involves more than 90% of the skin with erythroderma or T3, generalized lymphadenopathy and neoplastic T-cells, coined Sézary cells, in skin, lymph nodes and peripheral blood [2, 3]. While most patients present with *de novo* SS or primary SS, patients meeting the criteria for SS but with past history of MF are also called as secondary SS [2].

The International Society for Cutaneous Lymphoma (ISCL) and the European Organization of Research and Treatment of Cancer (EORTC) have provided consensus staging criteria to evaluate the blood burden in CTCL which are especially useful for the diagnosis of patients with erythroderma [3]. B0 stage or absence of blood involvement correspond to 5% or less Sézary cells; B1 or low blood burden is defined as more than 5% of Sézary cells but either less than 1000 Sézary cells /μL or absence of a clonal rearrangement of the T-cell receptor genes or both; B2 or leukemic phase is defined by clonal rearrangement of the TCR in the blood and either more than 1000 Sézary cells/μL or one of two of the following phenotypic criteria (1) expanded CD4+ or CD3+ cells with CD4/CD8 ratio of 10 or more (2) expanded CD4+ cells with abnormal phenotype including loss of CD7 (>40%) or CD26 (>30%) [3]. Recently, these cutoff levels were reevaluated in a multicenter study at 40% for CD7 loss and 80% for CD26 loss achieving a 83% sensitivity and 100% specificity to distinguish patients with SS from patients with erythrodermic inflammatory dermatoses [4]. Other phenotypic biomarkers such as CD158k expression in more than 5% of CD4+ cells [5] had only 33% sensitivity and 95% specificity [4]. Genetic biomarkers such as *MYC* gain at 8q24.21 (40%) and *MNT* loss at 17p13 (30%) were found useful along with as the demonstration of upregulated expression of *STAT4*, *TWIST1* and *DNM3* or *PLS3*, especially for cases with erythroderma and lack of abnormal phenotype [4].

The presence of an identical circulating and cutaneous T-cell clone was found to be associated with the diagnosis of CTCL in most patients having erythroderma [6]. The presence of a clonal T-cell population in the peripheral blood but not in the skin has *per se* limited diagnostic or prognostic value as it can be observed in reactive conditions, especially in the elderly [6–8]. Differentiating SS from clinical mimickers such as drug induced erythroderma or erythrodermic inflammatory dermatoses is crucial to formulate an effective diagnosis and appropriate therapy (for a commentary see [9])

In patients with MF or SS, we and others observed that the presence of an identical clone in skin and blood specimens is an independent and adverse prognostic factor whatever the histological subtype and disease stage [10–12]. However, there are general difficulties of statistical interpretations of biological or genetic parameters in patients with CTCL, especially when MF and SS cases are mixed together as a single entity.

Therefore, the demonstration of additional phenotypic and/or genetic alterations is necessary to assess blood involvement in CTCL and the malignancy of circulating monoclonal T-cells [13, 14].

Cytogenetic analysis using array comparative genomic hybridization (aCGH) have identified a specific cytogenetic profile of SS cells [15–17]. Indeed, 17p loss involving the *TP53* gene was observed by several groups in patients with SS but appeared rare in patients with MF except in cases with large cell transformation [15, 17–20]. These studies detected genetic copy number variation (CNV) with losses of genes such as *CDKN2A*, *E2A*, *A20* or *TP53* being more frequent than gains such as those *of MYC* [15, 17, 21].

Recent next generation sequencing (NGS) analysis of CD4+ selected blood cells in patients with SS have evidenced a variability of genetic aberrations resulting in a unique combination per patient of chromosomal imbalances, fusion genes and point mutations [22–25, 26, 27]. Interestingly, *TP53* alterations (mutation and/or copy number loss) were found as the most common genetic abnormality in patients with SS in several studies [24–26]. However, in the largest series of 101 SS cases studied so far [28], *TP53* mutation or deletion were detected in only 15% and 6% of patients, respectively. Indeed, the prevalence of *TP53* mutations was found highly variable ranging from 15% to 43% of cases by most exome sequencing studies (for a review see [22]). A first pivotal focused study identified a heterozygous loss of *TP53* was detected in 8 out of 9 patients with SS but *TP53* deleterious mutations were only detected in 3 out of 8 patients using Sanger sequencing [29]. Previous studies did not determine if *TP53* alterations were present at onset of the disease or detected in patients under treatment or during progression. The aim of the present study was to evaluate the incidence of *TP53* mutations in patients with primary and secondary SS at onset and during the course of the disease. Therefore, NGS analysis of *TP53* status was performed on unselected peripheral blood mononuclear cells and in skin specimens in the same patients. Then, we analyzed whether *TP53* mutations were associated with specific clinical features such as age, past-history of MF or evolutive features such as tumor formation and/or large cell transformation. We also evaluated *TP53* status along with several biological parameters such as the amount of Sézary cells (> or < 1000 cells/mm^3^), the presence of *TP53* genetic loss as determined by FISH analysis, and the length of telomeres.

## Materials and methods

### Patients and material

The cohort contained peripheral blood samples of 35 patients with SS at B2 stage according to the ISCL/EORTC criteria. According to the French Public Health and Bioethical Law, the present study was considered by our research direction lawyer as a non-interventional study without the need for ethics committee approval (Article L1121-1 and Article R1121-3). Moreover, informed consent was given to all patients with written consent for the use of the clinical data and research on their biological material. All data were collected anonymously.

In the 35 patients with SS, peripheral blood mononuclear cells (PBMC) were isolated using Pancoll (PAN-Biotech, Aidenbach, Germany) according manufacturer’s recommendations at SS diagnosis stage prior to treatment and for 28 patients during follow-up with a total of 63 analyzed blood samples. In patients with *TP53* mutation within blood samples, we also analyzed the initial skin specimen either at SS diagnosis time in 7 patients with primary SS and at MF stage in the 3 patients with secondary SS. Genomic DNA was extracted using standard phenol/chloroform protocol from peripheral blood mononuclear cells or frozen skin specimens.

A control group of 8 patients with idiopathic chronic inflammatory erythroderma was referred to the Dermatology department. There was neither evidence of drug induced erythroderma nor history of atopic dermatitis or psoriasis. Skin biopsy showed non-specific features and both PBMC count with phenotypic analysis and clonality analysis were performed for differential diagnosis with SS. In five patients, a paired analysis of skin and blood specimens was performed while in three patients only a skin specimen was evaluated for *TP53* status.

### Flow cytometry analyzes and sorting

In peripheral blood monuclear cells, TCR Vβ was determined using flow cytometry with the IOTest Beta Mark TCR Repertoire Kit (Beckman Coulter, Marseille, France) designed to identify 24 distinct TCR Vβ families according to manufacturer’s recommendations. Data acquisition was performed using a BD FACSCANTO II flow cytometer and analyzed using BD FACSDIVA^™^ software (BD Biosciences, le Pont de Claix, France). Tumor and non-tumor populations were sorted based on TCR Vβ22, CD4 and CD8 expression using BD FACSAriaTM II cell sorter (BD Biosciences).

### Targeted deep sequencing

Genomic mutation of *TP53* was studied by Next Generation Sequencing (NGS) using the Ion Ampliseq *TP53* panel (ThermoFisher Scientific, Life Technologies, Les Ullis France). The design covers 100% of the coding sequence of *TP53* including mutations at canonical splice sites and generates 24 amplicons on a length of 1 280 bases. Libraries were amplified by emulsion PCR and enriched using automatic system IonChef (ThermoFisher Scientific). Ion sphere particles were then sequenced with the Ion torrent personal genome machine (ThermoFisher Scientific) on 316v2 or 318v2 Chips (ThermoFisher Scientific) with high mean coverage > 3000X. Torrent Suite^™^ version 5.0 software (ThermoFisher Scientific) was used to perform data analysis. Reads were mapped to the human hg19 reference genome. Data processing, alignment and mutation calling were performed using the Torrent Suite^™^. The Variant Caller detected point mutations with a variant frequency ≥2% for Single Nucleotide Variation (SNV) and ≥5% for short insertion/deletion (INDEL). VCF files generated by Variant Caller were annotated by ANNOVAR [30].

In the 10 patients with *TP53* mutation, BAM of wild-type samples for *TP53* were also checked using Alamut Software (Interactive Biosoftware, Rouen, France).

### *TCRG* gene rearrangement analysis

TCRG gene rearrangement was studied by using a GC-clamp multiplex PCR denaturing gradient gel electrophoresis (DGGE) as originally described and used in further reports [6, 12, 31]. The procedure allows a direct clonality assessment and comparison of clonal rearrangement detected in blood and skin samples as migration of clonal bands depends on size and sequence-specific denaturation [6, 12]. For comparison of tumoral and non tumoral flow sorted cells, we performed *TCRG* gene rearrangement analysis using a standardized Biomed-2 protocol with commercially available reagents (Invivoscribe, San Diego, CA, USA) according to the EuroClonality/BIOMED-2 guidelines [32]. GeneScanning of PCR products was performed on a Applied 3100 genetic analyzer (Life Sciences, Les Ullis, France).

### FISH analysis

*TP53* FISH assay was performed using Metasystems FISH XL P53 Deletion Probe (D-5017-025-RG, Altussheim, Germany) according to the manufacturer’s protocol. Unselected fresh blood cells isolated by gradient separation or after short-term culture were fixed in Methanol:Acetic Acid (3:1) were spread on SuperFrost Plus positively charged slides, pretreated in 2x SSC at 37°C, and passed through graded ethanol and air-dried, as described [33, 34]. Formalin-fixed sections were also used and pretreated, as described [35]. Slides were co-denaturated with probe at 75°C for 2 minutes and hybridized at 37°C overnight using an automated HyBrite co-denaturation oven (Abbott Molecular, Rungis, France). Slides were then placed in washing buffer 1 (2x SSC/0.1% NP40) at room temperature for 2 min to remove the coverslips, immersed in 73°C washing buffer 2 (0.4x SSC/0.3% NP40) for 2 min, then placed in washing buffer 1 at room temperature for 1 min, passed through graded ethanol, air-dried and mounted with Vectashield mounting medium with DAPI (Vector laboratories, CliniSciences, Nanterre, France). Slides were analyzed with an epifluorescence microscope equipped with appropriate single band-pass filters (Abbott Molecular). FISH analysis was performed by 2 trained lecturers. The slide was considered as interpretable if the following requirements were fulfilled: (1) bright and distinct signals (2) low background relatively free of fluorescent particles or haziness.

### Quantitative fluorescence *in situ* hybridization

Quantitative fluorescence *in situ* hybridization (Q-FISH) was performed as previously mentioned [34]. Briefly, cell nuclei were prepared and spread onto microscope slides. The hybridization was performed according to manufacturer’s instructions (DakoCytomation, Trappes, France) with minor modifications. Thus, after pre-treatment steps nuclei were denatured in formamide 70% in 2xSSC at 70°C, rinsed in cold ethanol series and air dried. Peptide nucleic acid (PNA) probe for telomere conjugated with Cyanine 3 (Cy3) dye, was denatured at 80°C for 5 min. Nuclei were hybridized at 30°C for 2 hours into a ThermoBrite system (Leica Microsystemes, Nanterre, France). Then, slides were rinsed, washed, air dried and mounted with Vectashield (Vector Laboratories) containing 4′,6-diamidino-2-phenylindole dihydrochloride (DAPI).

### Statistical analysis

Data were analyzed according to the number of dependent variables and to the nature of dependent and independent variables. In order to test the normality of our data the Shapiro-Wilk test was used. For non-parametric statistical analysis the Wilcoxon-Mann Whitney or the Fisher’s exact test were used. This test is suitable for sample number below 30. To remove outlier values, Dixon’s test was applied. Survival was evaluated by Cox's proportional hazards analysis. The significance level was taken at P ≤ 0.05. The statistical tests were performed using the MedCalc software (Ostend, Belgium).

### Immunohistochemical for PD1, P53 and MDM2 expression

Immunohistochemical analysis was performed on fixed skin sections. The following primary antibodies and dilutions were used: Anti-p53 diluted 1:100 (Clone DO-7, DakoCytomation, Denmark), anti-PD1 diluted 1:50 (Clone MRQ-22 Zytomed, Diagomics, Blagnac, France) and anti-MDM2 diluted 1:50 (Clone IF2, ThermoFisher Scientific). Sections were incubated in 90°C 30mn/ER1 for P53, 20mn/ER2 for PD1 and 20mn/ER1 for MDM2. The analysis was performed using Bond-Max (Leica Microsystemes) according to the manufacturers’ recommendations. Both percentage of positive tumor cells and staining intensity (1+ to 3+) were recorded according to guidelines in an attempt to determine an H-score [36]. The predominant pattern (percentage x intensity) was finally recorded.

## Results

### Description of the cohort

Characteristics of the patients are summarized in Table 1 and S1 Table.

**Table 1:** Clinical and biological features of patients with primary Sézary Syndrome (SS) or with past-history of Mycosis Fungoides (MF) at secondary SS stage.

Characteristics	SS (n = 26)	Past-history of MF (n = 9)	Inflammatory erythroderma (n = 8)
**Male:female ratio**	1	0.5	3
**Median age at diagnosis (range),** years	75.5 (57–86)	62 (45–79)	74 (45–95)
**Mean delay between MF and SS,** months	/	41	/
**Erythroderma**	26	9 (7^a^)	8
**Sézary cell count ≥1G/L**	24	9	0
**T-cell clone peripheral blood**	26	9 (8^a^)	1
**Identical T-cell clone in blood and skin**	26	9	0

^a^Value corresponds to patients with secondary SS at MF initial diagnosis stage.

After review of initial and current stage, 26 patients presented with primary SS and 9 patients with secondary SS had a past-history of MF, mostly erythrodermic (n = 7). Patients with primary SS had a median age of 75.5 years at diagnosis and gender ratio (M/F) was 1. For patients with a past-history of MF or secondary SS, median age at MF diagnosis was 62 years and the gender ratio was 0.5. Mean and median follow-up of patients with primary SS were 52.0 and 45.5 months, respectively. Mean and median follow-up of patients with secondary SS were 70.0 and 50.0 months, respectively. In all patients, one blood sample at SS diagnosis was analyzed for *TP53* mutation as well as for *TCRG* gene rearrangement showing a monoclonal rearrangement (S1 Table). In the control group, median age was 74 and the gender ratio was 3.

### Detection of somatic *TP53* mutations in peripheral blood mononuclear cells of patients with SS

**Fig 1:**
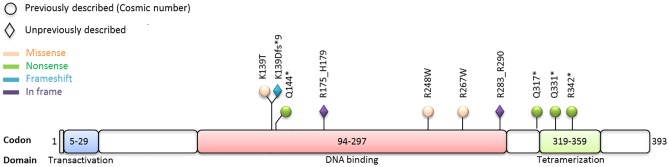
Schematic representation of somatic mutations identified in *TP53* by targeted deep sequencing (n = 35). Mutation sites were marked and amino acid changes were indicated. Colors and shapes indicated the kind of mutation and the presence in the COSMIC database.

We performed targeted deep sequencing of all coding and intron-exon flanking sequences of the *TP53* gene and studied 63 blood samples of 35 patients with SS using the Personal Genome Machine (PGM) and a commercially available NGS *TP53* panel kit. Somatic *TP53* mutations were identified in the blood of 10 out of 35 patients (29%) corresponding to 4 nonsense SNVs, 3 missense SNVs, 2 in-frame INDELs, and 1 frameshift INDEL. Interestingly, each case exhibited a single and unique mutation (Fig 1).

Among the 10 *TP53* mutations, two of them were present within the domain encoding for tetramerization property, and seven in the DNA-binding domain. Prediction analysis of Polyphen-2 (Polymorphism Phenotyping v2, http://genetics.bwh.harvard.edu/pph2/) showed a score greater than 0,96 for the 3 missense mutations. The three variants with INDELs were found absent in the COSMIC v77 database. The 2 in-frame deletions of 5 and 8 Amino Acids involved the DNA-binding domain and the frameshift mutation resulted in a stop codon at the beginning of this domain.

### *TP53* mutation is present in clonal T-cells

For patient P3, we first analyzed TCR Vβ expression on PBMC using the panel of 24 monoclonal antibodies to TCR Vβ families. Such analysis identified the expansion of a restricted TCR Vβ22+ CD4+CD8- population and these cells were isolated from other PBMC using flow cytometry cell sorting (Figure A in S1 Fig). After DNA extraction, we performed both clonality analysis of *TCRG* gene rearrangement and targeted deep sequencing of the *TP53* gene (Figure B in S1 Fig). The same *TP53* mutation as in the original unselected PBMC sample was detected in the TCR Vβ22+ CD4+CD8- sorted population with a 99% allelic frequency while a wild-type status was observed in the other sorted PBMC cells (Figure C in S1 Fig). This was correlated with the detection of a monoclonal TCRG rearrangement in the TCR Vβ22+ CD4+CD8- sorted cells and a polyclonal profile in the others (Figures A, B and C in S1 Fig).

### *TP53* mutations can be reliably detected in sequential blood specimens and in the skin of patients with SS

We analyzed 28 sequential blood samples from patients with *TP53* mutation in the blood. We also analyzed 7 skin samples of patients with primary SS and *TP53* mutation as well as 3 skin samples in patients with secondary SS. All BAM files of wild-type samples of these patients were checked using Alamut Software (Interactive Biosoftware, Rouen, France) in order to detect very low frequency mutations.

The same and unique mutation was found both in skin and blood samples in the 10 cases with *TP53* mutation. In these patients, sequential blood samples collected after treatment showed the persistence of the same *TP53* mutated clone. In six of these 10 patients and also in nine patients without mutation, sequential blood samples were collected after extracorporeal photophoresis and no additional *TP53* mutation was observed. Altogether, the incidence of *TP53* mutation was equivalent in patients who ever received UVA irradiation or not.

Several whole exome sequencing analysis reported a high rate of C>T transition in SS point mutations suggesting a causal role for UV exposure [25, 37]. In our cohort, 6 out of 7 point mutations corresponded to a C>T transition. However, such C>T transitions were not observed at NpCpC sites which correspond to canonical motifs modified after UVB exposure but occurred at NpCpG sites in 3 out of these 6 cases which has been attributed to a senescence-related process [38].

### Polymorphism P72R or P72P of the *TP53* gene

A specific *TP53* polymorphism Pro72-to-Arg (P72R) was suggested to be associated with cancer susceptibility or aggressiveness [39]. Indeed, this SNP was found homozygous or heterozygous in 5 out of 6 SS patients with wild-type *TP53* status [29]. Alternatively, Mc Girt et al observed a significant increase in the frequency of Pro72Pro (P72P) in a cohort of patients with MF and suggesting that this polymorphism could contribute to MF susceptibility in patients at a younger age than those with SS [40]. We therefore evaluated the presence of these single nucleotide polymorphisms (SNP) both at the diagnosis and during follow-up in SS samples. In the 35 patients, 22 were homozygous Arg72Arg (63%), 12 were heterozygous Pro72Arg (34%), and one was homozygous Pro72Pro (3%). There was no difference between our patients and the general population for the allelic frequency for this SNP: Arg72Arg (56%), Pro72Arg (37%), Pro72Pro (7%). When focusing on the 9 patients with MF and then secondary SS, we observed a slight increase of the homozygous Arg72Arg (78%) but such difference may result from the small size of this subgroup. In the control group of 8 inflammatory dermatoses, the distribution was similar with Arg72Arg (n = 6, 67%) and Pro72Arg (n = 2, 33%).

### *TP53* Copy Number Variation (CNV)

**Fig 2:**
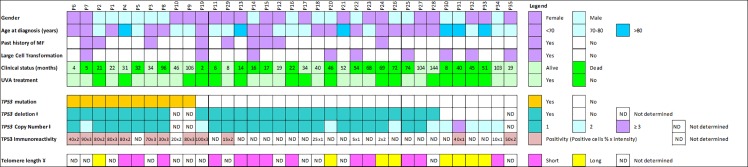
Clinical data and somatic alterations of *TP53* gene in Sézary syndrome. Age at diagnosis (years), Clinical status means survival time in months after diagnosis of Sézary syndrome until the death or last clinical status, ⱡ data determined by fluorescence *in situ* hybridization, ¥ data determined by quantitative fluorescence *in situ* hybridization.

Blood samples of 32 patients with SS were analyzed by FISH for *TP53* locus. No material was available in three patients including two patients with *TP53* mutation. FISH analysis showed a recurrent deletion of *TP53* in 27 out of 32 patients (84%) (Fig 2). In 8 of 10 patients with *TP53* mutation, a deletion of the *TP53* locus of at least one allele was observed. Two of the 8 cases exhibited 3 or 4 centromeric signals for chromosome 17 with loss of one or two *TP53* alleles, respectively. Six of these 8 patients showed a diploid pattern with loss of one *TP53* allele associated with mutation of the remaining allele suggesting a double knock-out.

Altogether, an imbalance and/or mutation of *TP53* were detected in 27 out of 32 patients who were analyzed both by NGS and FISH analyses (84%).

### Clinical and biological features associated with *TP53* alterations

Patients with *TP53* alterations (mean age 68) were younger than patients without *TP53* alteration (mean age 82) (p = 0.0075, Mann-Whitney test). We also analyzed survival according to *TP53* status but found no statistical difference. Survival was however shorter in patients with previous history of MF than in patients with primary SS whatever the *TP53* status (28 months versus 37 months, respectively, p = 0.0038) (S2 Fig).

When studying the number of circulating Sézary cells (less or more than 1000/μL, phenotypic criterion used for B2 staging (loss of CD7 and/or CD26), large-cell histological transformation, no specific feature was observed according to *TP53* status (Fig 2). However, large cell transformation was associated with worse prognosis (p = 0.0075) (S2 Fig).

When studying telomere length by quantitative FISH and q-PCR as reported previously [34], *TP53* alterations were more frequently observed in cases with shorter telomeres than in cases with longer telomeres (p<0.05, Fisher exact test) (Fig 2 and S3 Fig).

### P53 expression

We analyzed p53 and mdm-2 expression in tumoral cells in 17 skin samples of patients with SS by visual comparison with PD1 staining of SS cells to minimize counting of non-tumoral cells (data not shown). Indeed, p53 protein level was found increased by abnormal stabilization of the mutated form and sometimes associated with mdm-2 protein overexpression in CTCL cases [41, 42]. The cutoff used for IHC positivity was an immunoreactivity of more than 30% of tumoral cells but we also evaluated staining intensity as reported for individual cases (Fig 2). In patients with *TP53* mutation, p53 expression was observed in 89% of cases (8/9). Five of these cases presented a double *TP53* alteration (one mutated allele and one deleted allele) but were stained for p53 in 30–80% of tumour cells. Only one sample was negative corresponding to the patient with *TP53* frameshift mutation and deletion. We also observed p53 immunostaining in 44% of the patients (4/9) with *TP53* wild-type status. To explore whether p53 stabilization could be associated with the expression of the key negative regulator mdm-2, we analyzed immunostaining for mdm-2 in 14 skin biopsies. None of them was found positive (data not shown).

### *TP53* alterations are not detected in the skin and blood of 8 patients with chronic erythrodermic inflammatory dermatoses

Five pairs of blood and skin samples and three skin samples of 8 patients with reactive erythroderma were studied by Next Generation Sequencing (NGS) using the Ion Ampliseq *TP53* panel. No *TP53* mutation was detected.

Clonality analysis of *TCRG* gene rearrangements showed a polyclonal profile in samples of 4 patients (skin analysis in one patient, parallel blood and skin analysis in 3 patients). A monoclonal TCRG gene rearrangement was detected in samples of 4 patients (only at the skin level in 3 patients, both at the skin and blood level in one patient but with a different monoclonal band).

Skin sections of 7 of these 8 patients with reactive erythroderma were subjected to FISH analysis along with skin sections of 2 patients with SS and *TP53* monoallelic loss at the blood level. Interestingly, no *TP53* loss was observed in the dermal lymphoid infiltrate of patients with reactive erythroderma (Figure A in S4 Fig). In the two patients with SS, the upper-dermal infiltrate contained more than 20% of cells with an allelic loss pattern corresponding to the one observed at the level of PBMC of the same patient (Figure B in S4 Fig).

## Discussion

Next generation sequencing (NGS) techniques provide novel tools to determine the genetic landscape of cancer subtypes and to examine in depth the presence of single gene mutations. Using DNA extracted from unselected PBMC for routine *TCR* gene clonality analysis, a *TP53* mutation was detected in 10 out of 35 of SS cases (29%). Such rate is in the range of those reported in the different whole exome sequencing series using purified CD4+ blood cells (for a review see [22]). A unique mutation per patient was detected underscoring the need of the genetic coverage achieved by NGS. Here, we also demonstrate the presence of *TP53* mutations before treatment in 9 of these 10 patients. Targeted deep sequencing (>3000X) also detected the same mutation at the skin level in all patients with SS. While *TP53* mutations have not been detected by NGS in patients with classical MF [23, 40], they have been observed in some patients with erythrodermic MF and a blood T-cell clone [41]. The presence of an identical *TP53* mutation at the skin and blood level in patients at erythrodermic MF stage evolving into secondary SS establishes a biological link with SS, as suggested by others [25, 27].

In SS, the pattern of *TP53* gene mutations was suggestive of a role for UV radiation as point mutations were C>T transition occurring at di-pyrimidine sites [25, 41]. This would support that SS cells may originate from skin resident cells while others suggested that they derive from central memory T-cells with circulating capacities [43]. Although our data are limited to a single gene analysis, we have not observed that C>T transitions occurred at NpCpC sites for the *TP53* gene. *TP53* driving mutations in such elderly patients with SS may rather result from aging than from UV exposure, as in chronic lymphoid leukemia (CLL) [44]. However, as reported in two patients with a double inactivated *TP53* clone [26], we observed a relative younger age for patients with *TP53* alterations (mutation and/or deletion) than without.

Strikingly, *TP53* mutations have been detected at the skin level not only in B and T–cell cutaneous lymphomas but also in indolent lymphoproliferations such as lymphomatoid papulosis [41]. This strongly suggests that other cells such as keratinocytes may account for a mutated status when skin specimens only are analyzed. It also underscores the reliability of our parallel testing of blood and skin specimens for *TP53* status assessment.

At the biological level, the *TP53* mutations reported here were found to mainly affect the DNA binding or tetramerization domain of *TP53* [45]. By prediction analysis, they were found deleterious with a high probability score. As recently shown in breast cancers, NGS represents an unequivocal tool for the detection of mutations confirmed as mutant by the functional assay [46].

Using a simple FISH analysis on either unselected PBMC or skin sections, we also found the presence of at least one allelic alteration of *TP53* in 83% of SS cases. By comparison with patients with inflammatory erythroderma, we provide here evidence that TP53 alterations may be used to assess malignancy of circulating cells in patients with either erythroderma or a history of MF. Since the submission of our manuscript, another group reported the use of a panel of 11 FISH probes for the assessment of leukemic CTCL including SS, Transformed MF, Follicular-MF and MF [14]. Such customized panel was designed according to a recent exome study of selected CD4+ cells and evaluated against criteria established by the same group for leukemic evaluation in CTCL [27, 47]. Interestingly, *TP53* loss was a constant finding in their 10 SS cases [14]. Using international criteria to define primary and secondary SS, our study strongly supports that *TP53* assessment by FISH may be a robust tool for leukemic assessment of CTCL. This approach is also applicable to skin specimens for the differential diagnosis with inflammatory erythroderma.

The *TP53* tumor suppressor gene is involved in maintaining genomic stability and mediating response of cancer cells to therapy. In CLL, *TP53* inactivation is associated with complex karyotypes at onset of the disease providing a fitness advantage under therapy [44]. Similarly, most of our SS cases with *TP53* alteration displayed complex karyotypes (our personal data) and copy number alterations [17]. No correlation between *TP53* status and prognosis or large cell transformation was observed which may depend on other genetic events such as *MYC* gain or *CDKN2A* deletion [21, 48]. In SS cell lines such as SeAx, Hut78 and HH, *TP53* mutations were previously found associated with functional resistance to nutlin-3, an mdm-2 antagonist [29]. Whether *TP53* alteration may impact therapeutic response should be evaluated along with other genomic features in prospective trials.

SS mutational landscape has been reported to be complex with high inter-individual variability, especially in the generation of original fusion genes per patient [25, 26, 28]. *TP53* alteration in SS not only represents the most common genetic feature but may have a pivotal role in disease initiation as suggested by the early detection at diagnosis. Moreover, *TP53* alterations were found to correlate with telomere shortening in our patients. Such association may allow Sézary cells to escape senescence or checkpoint control after telomere crisis generating diverse chromosomal breaks and imbalances.

Altogether, *TP53* alterations were detected by targeted NGS or FISH analysis in 29 out of 35 patients with SS (83%) and this incidence was slightly higher in the 32 cases analyzed by both techniques (84%). Therefore, either FISH alone or a combined analysis with NGS may be a valuable tool for the diagnosis of patients with erythroderma and a circulating monoclonal T-cell population, especially in cases lacking the phenotypic criteria proposed by the ISCL/EORTC [3]. Both phenotypic and genotypic criteria will probably replace the morphological criterion of Sézary cell count which lacks reproducibility [47]. Although less recurrent genetic imbalances such as *MYC* gain (40%) and *MNT* loss (30%) together with variable genomic or epigenetic criteria have been proposed to differentiate SS from erythrodermic inflammatory dermatoses [4, 49], our study is original in supporting the introduction of a simple and robust *TP53* testing in algorithms for the diagnosis of patients with erythroderma as well as for leukemic assessment in patients with CTCL.

## Supporting information

S1 Table Patient features and histopathological data. * for patients with large cell transformation.
(DOCX)

S1 Fig Flow cytometry, clonality analysis and TP53 assessment demonstrate the presence of TP53 mutation in TCRVβ22+ clonal T-cells. A: Flow cytometry of patient P3 peripheral blood mononuclear cells (PBMC) (left column) followed by cell sorting of TCRVβ22+ tumor cells (median column) and non-TCRVβ22+ cells (right column). B: TCRG gene rearrangement analysis by the BIOMED-2 gene scanning assay C: Results of targeted deep sequencing of the *TP53* gene (The presence of an identical *TCRG* monoclonal rearrangement in total PBMC and sorted TCRVβ22+ tumor cells correlates with the presence of an identical *TP53* c.991C>T nonsense mutation while non-TCRVβ22+ sorted cells exhibit a polyclonal profile and a wild-type *TP53* status.
(TIF)

S2 Fig Survival analysis according to Cox's proportional hazards analysis between patients with primary or secondary Sézary syndrome (A) and patients with or without large cell transformation (B). (TIF)

S3 Fig Correlation between telomeres length and *TP53* status according to Fisher’s exact test. The length of telomeres was determined by quantitative fluorescence *in situ* hybridization (FISH). *TP53* status was assessed both by FISH and targeted deep sequencing.
(TIF)

S4 Fig Fluorescence *in Situ* Hybridization (FISH) analysis of skin sections of patients with either inflammatory dermatoses or Sézary syndrome. A:. A balanced and diploid FISH pattern after hybridization with the locus specific *TP53* probe (red signals) and the control chromosome 17 probe (green signals) is observed in inflammatory cells of the upper dermis in one patient with inflammatory erythroderma. B: A monoallelic *TP53* deletion with loss of one red signal corresponding to the *TP53* locus is detected in Sézary cells (arrows) present in the upper dermis in one patient with Sézary syndrome.
(TIF)

## Abbreviations

CTCL: cutaneous T-cell lymphoma
EORTC: European Organization of Research and Treatment of Cancer
FISH: Fluorescence *in situ* hybridization
INDEL: Insertion/deletion
ISCL: International Society for Cutaneous Lymphoma
MF: Mycosis Fungoides
NGS: Next Generation Sequencing
PBMC: Peripheral Blood Mononuclear Cells
SNP: Single Nucleotide Polymorphism
SNV: Single Nucleotide Variant
SS: Sézary syndrome
