# Supplementary material for: TP53 alterations in primary and secondary Sézary syndrome: A diagnostic tool for the assessment of malignancy in patients with erythroderma
Source: PLoS One. 2017 Mar 16;12(3):e0173171. doi: 10.1371/journal.pone.0173171 (PMC5354275; doi:10.1371/journal.pone.0173171)
Supplement: S1 Table — * for patients with large cell transformation. (DOCX) [file pone.0173171.s001.docx]

| **Patient ID** | **Gender** | **Age at diagnosis** | **Past-history of MF** | **TNM (MF)** | **TNM (SS)** | **Number of Blood samples** | **Skin biopsy** |
| --- | --- | --- | --- | --- | --- | --- | --- |
| P7 | F | 64 | Yes | T2bN0M0 B0b | *T4N1M0 B2 | 2 | Yes |
| P19 | F | 49 | Yes | T2N0M0 B0b | *T4N1M0 B2 | 2 |  |
| P3 | M | 45 | Yes | T4N0M0 B0b | T4N0M0 B2 | 2 | Yes |
| P15 | F | 57 | Yes | T4N0M0 B0b | T4N0M0 B2 | 2 |  |
| P2 | M | 70 | Yes | T4N0M0 B1b | T4N1M0 B2 | 2 | Yes |
| P12 | F | 79 | Yes | T4N0M0 B1b | T4N0M0 B2 | 2 |  |
| P24 | F | 62 | Yes | T4N0M0 B1b | T4NxM0 B2 | 2 |  |
| P29 | F | 72 | Yes | T4N0M0B B0b | T4N0M0 B2 | 2 |  |
| P14 | M | 55 | Yes | T4N3M0 B0b | *T4(3)N3M0 B2 | 2 |  |
| P16 | M | 70 | No | / | T4N1M0 B2 | 1 |  |
| P8 | M | 69 | No | / | T4N1M0 B2 | 2 | Yes |
| P30 | M | 85 | No | / | *T4(3)N1M0 B2 | 1 |  |
| P4 | M | 83 | No | / | T4N2M0 B2 | 2 | Yes |
| P13 | F | 82 | No | / | T4N0M0 B2 | 1 |  |
| P21 | F | 86 | No | / | T4N0M0 B2 | 1 |  |
| P11 | F | 76 | No | / | T4N1M0 B2 | 1 |  |
| P18 | M | 64 | No | / | T4N0M0 B2 | 2 |  |
| P5 | M | 76 | No | / | T2NxM0 B2 | 2 | Yes |
| P1 | M | 66 | No | / | T4N1M0 B2 | 2 | Yes |
| P26 | M | 60 | No | / | T2NXM0 B2 | 2 |  |
| P10 | F | 78 | No | / | T4N0M0 B2 | 2 | Yes |
| P23 | F | 74 | No | / | T4N0M0 B2 | 2 |  |
| P34 | M | 78 | No | / | T4NxM0 B2 | 2 |  |
| P9 | F | 77 | No | / | T4N0M0 B2 | 2 | Yes |
| P20 | M | 69 | No | / | *T4(3)N3M0 B2 | 2 |  |
| P25 | M | 62 | No | / | *T3N0M0 B2 | 2 |  |
| P35 | F | 75 | No | / | *T4N0M0 B2 | 2 |  |
| P32 | F | 80 | No | / | *T4N0M0 B2 | 2 |  |
| P17 | F | 79 | No | / | T2aN0M0 B2 | 2 |  |
| P6 | F | 61 | No | / | T4N1M0 B2 | 2 | Yes |
| P31 | M | 82 | No | / | T4N0M0 B2 | 2 |  |
| P27 | F | 61 | No | / | T4N3M0 B2 | 2 |  |
| P28 | F | 57 | No | / | T4N0M0 B2 | 2 |  |
| P33 | F | 86 | No | / | T4N3M0 B2 | 1 |  |
| P22 | M | 65 | No | / | *T4N0M0 B2 | 1 |  |
