# Supplementary figures and images for: TP53 alterations in primary and secondary Sézary syndrome: A diagnostic tool for the assessment of malignancy in patients with erythroderma
Source: PLoS One. 2017 Mar 16;12(3):e0173171. doi: 10.1371/journal.pone.0173171 (PMC5354275; doi:10.1371/journal.pone.0173171)

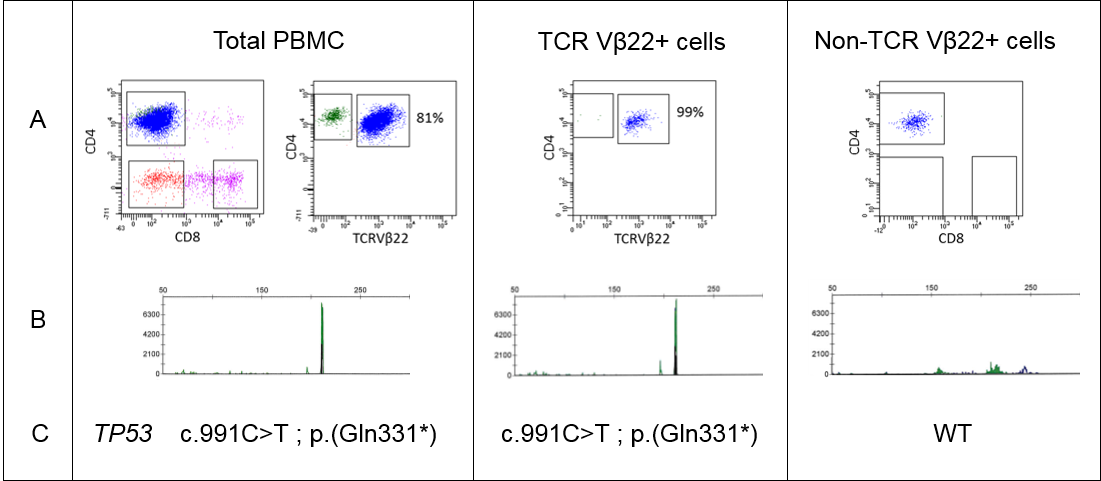

Supplement: S1 Fig — A: Flow cytometry of patient P3 peripheral blood mononuclear cells (PBMC) (left column) followed by cell sorting of TCRVβ22+ tumor cells (median column) and non-TCRVβ22+ cells (right column). B: TCRG gene rearrangement analysis by the BIOMED-2 gene scanning assay C: Results of targeted deep sequencing of the TP53 gene (The presence of an identical TCRG monoclonal rearrangement in total PBMC and sorted TCRVβ22+ tumor cells correlates with the presence of an identical TP53 c.991C>T nonsense mutation while non-TCRVβ22+ sorted cells exhibit a polyclonal profile and a wild-type TP53 status. (TIF) [file pone.0173171.s002.tif]

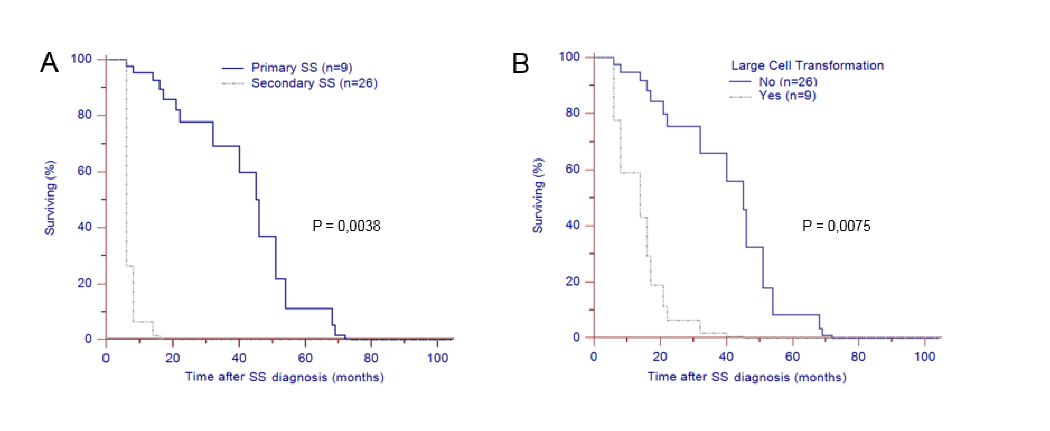

Supplement: S2 Fig — (TIF) [file pone.0173171.s003.tif]

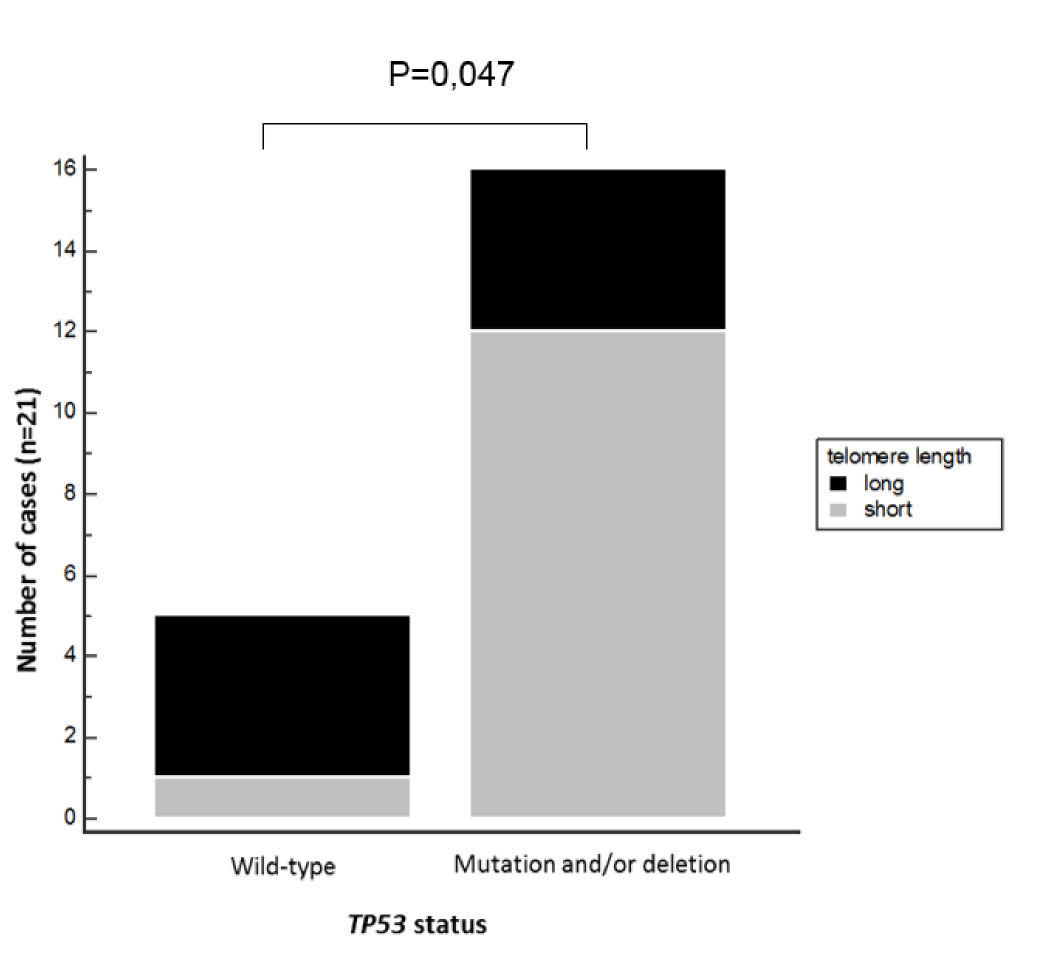

Supplement: S3 Fig — The length of telomeres was determined by quantitative fluorescence in situ hybridization (FISH). TP53 status was assessed both by FISH and targeted deep sequencing. (TIF) [file pone.0173171.s004.tif]

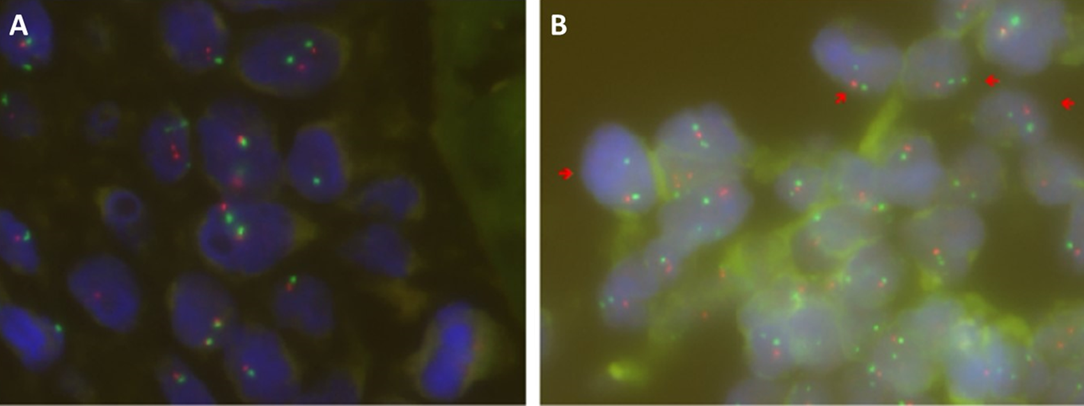

Supplement: S4 Fig — A:. A balanced and diploid FISH pattern after hybridization with the locus specific TP53 probe (red signals) and the control chromosome 17 probe (green signals) is observed in inflammatory cells of the upper dermis in one patient with inflammatory erythroderma. B: A monoallelic TP53 deletion with loss of one red signal corresponding to the TP53 locus is detected in Sézary cells (arrows) present in the upper dermis in one patient with Sézary syndrome. (TIF) [file pone.0173171.s005.tif]
